# Performance of leflunomide as a steroid-sparing agent in giant cell arteritis: A single-center, open-label study

**DOI:** 10.3389/fmed.2022.1069013

**Published:** 2022-11-10

**Authors:** Jelka Kramarič, Žiga Rotar, Matija Tomšič, Alojzija Hočevar

**Affiliations:** ^1^Department of Rheumatology, University Medical Centre Ljubljana, Ljubljana, Slovenia; ^2^Faculty of Medicine, University of Ljubljana, Ljubljana, Slovenia

**Keywords:** giant cell arteritis, open-label study, leflunomide, relapses, steroid sparing

## Abstract

**Background:**

The management of giant cell arteritis (GCA) remains challenging and many patients require prolonged glucocorticoid treatment due to high disease relapse rates. We aimed to evaluate the role of leflunomide as a steroid-sparing agent in GCA.

**Methods:**

This prospective open-label study included patients diagnosed with GCA between July 2014 and August 2020 and followed them for 96 weeks. At the time of diagnosis all patients received treatment following a predefined glucocorticoid regimen. At week 12 of follow-up, 10 mg of leflunomide per day was recommended as an adjunctive therapy. The decision to start with leflunomide treatment was patient-dependent. Follow-up visits were performed adhering to a predetermined protocol. The number of relapses, the cumulative glucocorticoid dose and treatment-related adverse events were recorded and compared between glucocorticoid-only and leflunomide groups.

**Results:**

Of the 215 GCA patients [67.6% female, median (IQR) age 74 (66–79) years], 151 (70.2%) received leflunomide at week 12 (leflunomide group); the others continued with glucocorticoids (glucocorticoid-only group). During the study 64/215 (29.8%) patients relapsed. Of the 51 patients who relapsed after 12 weeks, 22/151 patients (14.6%) and 29/64 patients (45.3%) were in the leflunomide and glucocorticoid-only group, respectively (*p* = 0.001; NNT 3.3 for leflunomide). Furthermore, 80/151 patients in the leflunomide group managed to stop glucocorticoids at week 48 [with relapses in 6/80 patients (7.5%)]. The cumulative glucocorticoid dose was lower in the leflunomide group (*p* = 0.009).

**Conclusion:**

In our cohort, leflunomide safely and effectively reduced the GCA relapse rate and demonstrated a steroid-sparing effect in over three quarters of patients.

## Introduction

Giant cell arteritis (GCA) represents the most common primary vasculitis of large and medium-sized arteries in the population aged over 50 in Europe and North America (1). It is a rheumatologic emergency and as such requires prompt anti-inflammatory treatment to prevent irreversible ischemic complications (2–4). Glucocorticoids remain the cornerstone of treatment due to their rapid onset of action (5). Unfortunately, almost half of patients relapse during glucocorticoid tapering, and around half after glucocorticoid withdrawal (4, 6). Therefore, many patients need prolonged treatment resulting in high cumulative glucocorticoid doses (5). Therefore, patients are at risk of developing glucocorticoid-related adverse events and complications such as diabetes, arterial hypertension infections, osteoporosis, fractures and steroid myopathy (7). Many conventional synthetic and biologic disease-modifying anti-rheumatic drugs (csDMARDs, bDMARDs) have been studied for their steroid-sparing effect in treating GCA. A superior efficacy compared to glucocorticoids, as well as reduced cumulative glucocorticoid exposure and increased rate of sustained remission was compellingly shown only for tocilizumab (8). However, bDMARDs are contraindicated in some patients and are associated with a significant cost. Among csDMARDs, methotrexate is recommended as an alternative, despite the very modest evidence supporting its use (9, 10). Nevertheless, the use of methotrexate is contraindicated in chronic kidney disease, which is relatively common in the elderly population, which is the population most often affected by GCA.

Leflunomide is a safe and effective csDMARD for the treatment of inflammatory arthritides as well as systemic vasculitides (e.g., granulomatosis with polyangiitis and Takayasu arteritis) (11, 12). Due to its mechanism of action the potential effectiveness of leflunomide is expected in GCA, as it suppresses the production of proinflammatory cytokines through the activation of dendritic cells and also weakens the action of the T-cell response (13, 14).

There are no randomized controlled clinical trials supporting the efficacy of leflunomide as a steroid-sparing agent in GCA, but data from a few single-center studies, case series and case reports are promising (15–21). Our center reported in 2019 a study on leflunomide in GCA patients, comparing 30 patients treated with leflunomide vs. 46 on glucocorticoids (15). In the current extended study (both in the number of patients and the study period) we evaluated the effectiveness of leflunomide in the largest cohort of patients with GCA reported up-to-date.

## Methods

### Setting

This prospective open-label study was performed at the Department of Rheumatology, University Medical Center Ljubljana, a secondary/tertiary level teaching hospital, where we manage most GCA cases from the region using our fast-track protocol (4).

### Patients

In the present study we enrolled patients diagnosed with GCA between July 2014 and August 2020.

GCA diagnosis was based on the corresponding clinical and laboratory features and either the positive result of a temporal artery biopsy as defined by the 1990 American College of Rheumatology criteria for the classification of GCA (22) and/or the positive result of imaging [color Doppler sonography of seven arterial territories–paired temporal, facial, occipital, carotid, vertebral, subclavian and axillary, or positron emission tomography/computed tomography (PET/CT) with the use of 18F-fluoro-2-deoxy-D-glucose (18F-FDG)].

### Baseline evaluation and follow-up

The baseline patient work-up included a thorough history of GCA symptoms, comorbidities, a complete physical examination, extensive laboratory tests and imaging (color Doppler sonography or 18F-FDG PET/CT) or a temporal artery biopsy.

Follow-up visits with predetermined clinical evaluation and laboratory tests were performed at 4, 12, 24, 48, 52 (± 2) and 96 (± 2) weeks after diagnosis. Additional unscheduled visits were arranged for patients who relapsed during glucocorticoid tapering or after glucocorticoid discontinuation.

Patients who completed all scheduled follow-up visits were included in the analysis.

Disease relapse was defined as the disease worsening or new disease activity after the initial remission. We subdivided the observed relapses into laboratory-only, clinical-only or clinical and laboratory. Other reasons for the observed symptoms and/or elevated inflammatory markers (i.e., infections, malignancy, other underlying disease) had to be excluded.

Clinical relapse was defined as the reappearance of signs of cranial ischemia (headache, yaw claudication, visual disturbances–usually objectivized by an ophthalmologist), constitutional symptoms (fever, weight loss, night sweating), symptoms of polymyalgia rheumatica, and in the case of limb ischemia, the worsening of the ischemia after initial improvement after treatment. The symptoms/signs have to improve after the intensification of immunomodulatory treatment.

In the laboratory we monitored the C-reactive protein and erythrocyte sedimentation rate. A persistent increase of C-reactive protein and erythrocyte sedimentation rate, after the exclusion of infection and other causes for elevation of inflammatory parameters (e.g., malignancy), that responded to the escalation of immunomodulatory treatment was documented as a laboratory GCA relapse.

We recorded the number of relapses during the first 96 weeks of treatment, the cumulative glucocorticoid dose for each patient at 96 weeks, and adverse events associated with glucocorticoids or leflunomide.

### Treatment protocol and patient stratification

The detailed study protocol has been already described (15). Briefly, according to EULAR recommendations, we initiated treatment with glucocorticoids in all patients at the time of GCA diagnosis (23, 24). The initial dose of oral methylprednisolone was 0.8 mg/kg of body weight once per day (qd), but no <32 mg qd and no more than 48 mg qd. Patients with cranial GCA experiencing ischemic complications such as visual disturbance and those with extracranial large vessel GCA additionally received methylprednisolone 250 mg intravenously for three consecutive days prior to receiving methylprednisolone orally.

The tapering of glucocorticoid therapy started after 2–4 weeks. The dose of methylprednisolone was reduced by 4 mg weekly to 16 mg qd, then 2 mg each other week to 8 mg qd, then 1 mg monthly to a maintenance dose of 4 mg qd. At week 48 we discontinued glucocorticoid treatment in patients in the leflunomide group who were in remission during the first 48 weeks of follow-up. Patients who chose to remain in the glucocorticoid-only group and patients in the leflunomide group with a relapse continued treatment with the lowest effective glucocorticoid dose after week 48.

At week 12 the add-on therapy with leflunomide 10 mg qd was offered to all patients without contraindications for leflunomide (e.g., liver failure, bone marrow suppression). Patients who refused treatment with leflunomide were allocated to the glucocorticoid-only group.

In cases of GCA relapse, the methylprednisolone dose was temporarily increased by 8–12 mg qd on top of the last previously effective dose and leflunomide (10 mg qd) was added to the treatment for patients in the glucocorticoid-only group. In cases of GCA relapse in patients who were in the leflunomide group, the methylprednisolone dose was increased as described above, and the dose of leflunomide was increased from 10 to 20 mg qd. In cases of active GCA, despite this intervention or in cases of adverse events attributable to leflunomide, leflunomide was substituted with oral methotrexate (15 to 20 mg weekly) or a bDMARD (tocilizumab or ustekinumab).

### Adverse events

Adverse events were systematically recorded with particular focus on 17 types of adverse events attributable to either glucocorticoids or leflunomide: steroid diabetes, steroid myopathy, osteoporotic fracture, cataract, glaucoma, severe infection (defined as a need for antibiotic treatment or hospital admission, including tuberculosis), hair loss, weight loss, diarrhea, significant increase in blood pressure (defined as the need to increase or institute antihypertensive therapy), elevated transaminases, skin bruises, skin rash, leflunomide induced pneumonitis, polyneuropathy and bone marrow toxicity.

### Ethical standards

The study was approved by the National Medical Ethics Committee, approval number 112/09/14.

All patients provided their written consent for the use of their demographic and clinical data.

### Statistical analysis

Descriptive statistics were used to analyse the studied population. The results were expressed as medians and interquartile ranges (IQR) for metric continuous variables with skewed distribution, and as numbers and proportions for categorical variables. To test the differences between the observed groups, we used the Mann–Whitney U test for metric and Fisher's exact test for categorical variables. The significance threshold selected in all analyses was set at 0.05. The Jamovi (Sydney, Australia) software (version 2.3.0) was used for statistical calculations.

## Results

### Stratification of GCA patients and baseline patient characteristics

**Figure 1:**
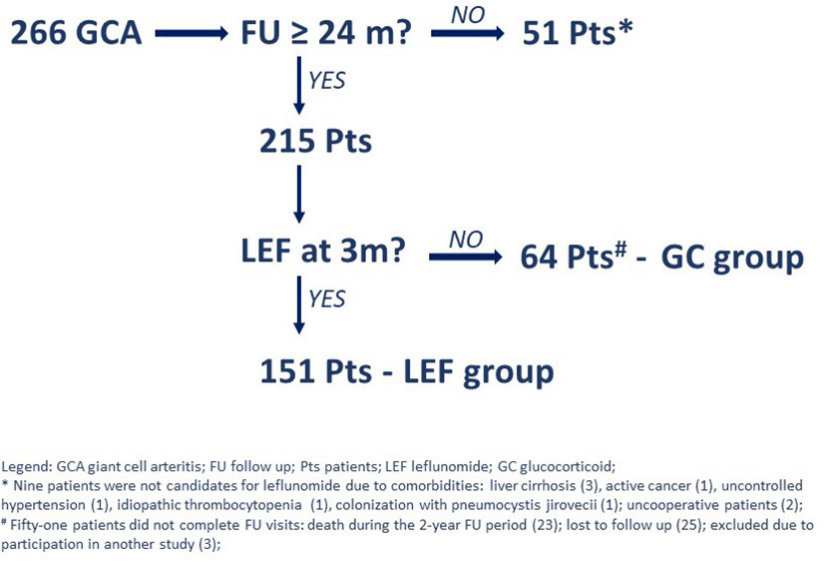
Flow chart in giant cell arteritis patients.

During the 74-month period, we identified 266 patients with newly diagnosed GCA, of whom 51 patients did not complete all the scheduled visits and were therefore excluded from further analyses. Of the remaining 215 patients, 151 (70.2%) chose to start leflunomide (i.e., leflunomide group) and 64 (29.8%) chose not to (i.e., glucocorticoid-only group) (Figure 1).

All patients underwent a vascular ultrasound, and 168/215 (78.1%) had a positive temporal artery ultrasound. In addition, 42 of 47 patients with a negative temporal artery ultrasound had ultrasound findings consistent with vasculitis in one of the other examined arteries. A temporal artery biopsy was performed in 100 patients, and was positive in 86 cases (86.0%). A PET/CT was performed in 27 patients and was consistent with vasculitis in 24 cases (88.9%).

The baseline demographic and clinical characteristics as well as inflammatory markers of the 215 GCA patients (67.6% female) who were followed for at least 96 weeks are presented in Table 1, column A. Their median (IQR) age was 74 (66–79) years. Cranial GCA was diagnosed in 138 (64.2%) patients and the rest had extracranial large vessel GCA. There were no significant differences in baseline demographic and clinical characteristics or inflammatory markers between the leflunomide and glucocorticoid-only group (Table 1, column B).

**Table 1:** Baseline characteristics of giant cell arteritis patients in the leflunomide and glucocorticoid-only group.

	**A**	**B**		
**Characteristics**	**ALL GCA**	**LEF**	**GC**	**p value**
	**(*n* = 215)**	**(*n* = 151; 70%)**	**(*n* = 64; 30%)**	
Female	146 (67.9%)	71.5	59.4	0.110
Age (years)	74 (66; 79)	73 (66; 78)	77 (69; 84)	0.177
Constitutional symptoms	161 (74.9%)	73.5	78.1	0.606
Polymyalgia rheumatica	31 (14.4%)	15.9	10.9	0.402
Headache	149 (69.3%)	71.5	64.1	0.332
Jaw claudication	95 (44.2%)	46.4	39.1	0.369
Visual symptoms	46 (21.4%)	19.9	25.0	0.467
Visual loss	13 (6.0%)	4.6	9.4	0.214
Stroke	4 (1.9%)	2.6	0	0.320
Large vessel vasculitis	77 (35.8%)	33.1	42.2	0.217
ESR (mm/h)	83 (60; 110)	80 (60; 110)	91 (60; 111)	0.610
CRP (mg/l)	91 (46; 140)	91 (47; 140)	95 (37; 137)	0.960

GCA, giant cell arteritis; LEF, leflunomide group; GC, glucocorticoid-only group; ESR, erythrocyte sedimentation rate; CRP, C-reactive protein. Results are presented as n (%), except age, ESR, and CRP which are presented as median (IQR).

### Follow-up from week 0 to week 96

During the study 64/215 (29.8%) patients relapsed. Overall, we documented 81 relapse episodes, as some patients had more than one relapse: we documented one relapse in 51 (79.7%) patients, two relapses in 10 (15.6%) patients, three relapses in two (3.1%) patients and four relapses in one (1.6%) patient).

Patients with extracranial large vessel GCA relapsed more frequently compared to cranial limited GCA (46.9% patients had large vessel involvement in the relapsing GCA group, compared to 31.1% cases of large vessel involvement in the non-relapsing GCA group, *p* = 0.031).

In 18 patients the first relapse occurred during the first 12 weeks after diagnosis (i.e., before adding the leflunomide), while 63 episodes occurred in 51 patients from week 12 to week 96 of the follow-up.

Of the 51 patients with a relapse after week 12 of follow-up, 22 patients were in the leflunomide group [22/151 patients (14.6%); 25 episodes] and 29 in the glucocorticoid-only group [29/64 patients (45.3%); 38 episodes]. The difference in the relapse rates between the groups was significant (*p* < 0.001), with the number needed to treat (NNT) for leflunomide standing at 3.3 (95% CI 2.3; 5.5). Among the documented relapses, 59% were laboratory-only, 6% were clinical-only and 35% were concurrently clinical and laboratory.

In 18 relapsing patients leflunomide was increased from 10 to 20 mg. In 14 patients, methotrexate was prescribed after relapse. In two relapsing patients ustekinumab was used and in one patient tocilizumab was used after leflunomide failure (however this patient was finally treated with secukinumab).

### Follow-up of leflunomide group after glucocorticoid withdrawal

In 80 (53.0%) of the 151 patients in the leflunomide group, glucocorticoid treatment was discontinued at week 48, as per protocol. Three patients decreased the methylprednisolone dose after 48 weeks from 4 mg qd to 2 mg qd. The rest continued treatment with methylprednisolone of 4 mg qd. During the follow-up period in the leflunomide group after glucocorticoid discontinuation (from week 48 to week 96) we documented relapse in 6 out of the 80 (7.5%) patients (these relapses were included in the quota of all relapses in leflunomide group).

### Cumulative glucocorticoid dose at week 96

At the last follow-up visit (week 96) the cumulative median (IQR) prednisolone-equivalent doses were 7.0 (5.2; 7.7) g and 7.7 (7.0; 7.9) g in the leflunomide and glucocorticoid-only group, respectively. The difference in cumulative glucocorticoid dose was significant (*p* = 0.009).

### Adverse events

We documented at least one of the adverse events of special interest in 187 (87.0%) patients. Adverse events were observed in 87.4 and 85.9% of patients in the leflunomide and glucocorticoid-only group, respectively. In total we observed 419 adverse events (Table 2, column A). The two adverse events that were significantly more frequent in the leflunomide group were hair loss (*p* = 0.016) and diarrhea (*p* = 0.016). None of the patients had leflunomide-associated bone marrow toxicity, pneumonitis or polyneuropathy. The frequencies of adverse events are shown in Table 2, column B.

**Table 2:** Adverse events in the leflunomide and glucocorticoid-only group.

	**A**	**B**		
**Adverse event**	**ALL GCA**	**LEF**	**GC**	**p value**
	**[*n* = 215; *n* (%)]**	**(*n* = 151; %)**	**(*n* = 64; %)**	
Bruises	119 (55.3%)	52.3	62.5	0.180
Steroid diabetes	61 (28.4%)	27.2	31.3	0.620
Steroid myopathy	49 (22.8%)	25.8	15.6	0.113
Osteoporotic fracture	10 (4.7%)	5.3	3.1	0.727
Cataracts	29 (13.5%)	11.9	17.2	0.382
Glaucoma	3 (1.4%)	0.7	3.1	0.212
Severe infection	43 (20.0%)	17.2	26.6	0.137
Hair loss	54 (25.1%)	29.8	14.1	0.016
Weight loss	14 (6.5%)	7.9	3.1	0.239
Diarrhea	19 (8.8%)	11.9	1.6	0.016
Increased BP	9 (4.2%)	4.6	3.1	1.0
Elevated transaminases	5 (2.3%)	2.6	1.6	1.0
Bone marrow toxicity	0	0	0	-
Leflunomide rash	1 (0.5%)	0.7	-	-
Tuberculosis	0	0	0	-
Leflunomide pneumonitis	0	0	-	-
Leflunomide neuropathy	0	0	-	-

GCA, giant cell arteritis; LEF, leflunomide group; GC, glucocorticoid-only group; BP, blood pressure.

Forty-one out of 151 (27.2%) patients discontinued leflunomide due to one or more adverse events, after a median (IQR) 18 (7, 27) weeks of treatment.

## Discussion

The management of GCA remains a challenge, despite new insights into disease pathogenesis, improved diagnostic options, fast-track protocols and approval of bDMARDs for its treatment. In spite of the growing choice of treatment options, glucocorticoids have remained the mainstay of therapy for GCA regardless of their long-term adverse effects and increased awareness of the importance of glucocorticoid-sparing treatment regimens. Among csDMARDs, methotrexate has been extensively studied in GCA but without much success (9, 25). Leflunomide, which is an effective and safe csDMARD, has not been extensively studied in GCA, even though its mechanism of action supports its potential benefit in the treatment of GCA due to its immunomodulatory effect through the inhibition of dendritic cell maturation, which is considered the principal pathogenetic mechanism in GCA (13, 14). Leflunomide also modulates interleukin-6 levels, known to be elevated in GCA (26).

To our knowledge, we have reported the largest cohort of patients who have cranial or large vessel GCA, were treated with an add-on therapy with leflunomide and followed for 96 weeks. We focused on the occurrence of relapses, assessing the potential steroid-sparing effect of leflunomide and its safety.

After week 12 of follow-up, i.e., after the cohort was split into leflunomide and glucocorticoid-only group, we observed significantly fewer relapses in the leflunomide group, with an NNT for leflunomide of ~3 patients. Furthermore, even after the glucocorticoids were discontinued at week 48 for more than half of the patients in the leflunomide group, only 7.5% of patients relapsed in the period from week 48 to week 96. This data demonstrates that most patients in whom glucocorticoid can be discontinued after 48 weeks remain in remission on leflunomide alone for at least a year. Additionally, these patients achieved and remained in remission with a significantly lower cumulative glucocorticoid dose at week 96. This effect was reached by adding a low-dose leflunomide of only 10 mg qd, which is lower than the standard dose for treatment of rheumatoid arthritis (27).

There were no serious or life-threatening adverse events observed that were attributable only to leflunomide, such as hypersensitivity reaction, bone marrow toxicity, pneumonitis or polyneuropathy. The rate of adverse events observed was similar between the two groups, since both groups received glucocorticoids. The two adverse events that were significantly more common in the leflunomide group were hair loss and diarrhea, which were resolved after discontinuation of leflunomide, suggesting that the risk of persistent and relapsing GCA and prolonged treatment with glucocorticoids outweighs the risk of leflunomide-associated toxicity. Moreover, there was no significant difference in the occurrence of severe infections between the groups, a finding further supporting the use of leflunomide. Numerically speaking, the infection rate was even lower in leflunomide group. We also found our results to be in line with a recent study in large vessel GCA, where a 24.3% discontinuation rate was reported (20). Similarly, in studies in rheumatoid arthritis, the drop-out due to leflunomide adverse events was 25% (28).

These data extend previous observations from the first ever prospective observational single-center study, conducted at our center, which confirmed a significant difference in the rate of relapses in the group receiving leflunomide in addition to standard glucocorticoid therapy compared to the control group receiving glucocorticoids alone during the first 48 weeks of follow-up (13.3 vs. 39.1%, *p* = 0.02), but with a lower number of enrolled patients (76 patients) (15).

A recent prospective Indian observational study reported 22 patients newly diagnosed with cranial-only GCA with an add-on therapy with leflunomide to a predefined glucocorticoid regimen at week 0, demonstrating that the maintenance of continuous steroid-free remission was achieved in 68% of patients for a median follow-up period of 24 months (18). Seven (31.8%) patients in this cohort experienced a clinical relapse after a median of 12 months after initial remission, a rate significantly higher than in our cohort; however, due to the different design of the study and limited number of patients, a comparison is inapplicable.

Another recent, though retrospective study from the UK reported long-term experience with the use of leflunomide in a cohort of 70 patients with large-vessel GCA (20). Of all the patients on leflunomide, 23% experienced at least one relapse; however, patients starting leflunomide due to a relapse later on in the course of the disease course were also included. Compared to our findings, we can speculate that the relapse rate might be lower if all patients were started on leflunomide early on in the course of the disease, even though our cohort included large-vessel as well as cranial GCA patients. The findings in this study were additionally supported by the use of imaging, confirming a positive response in the majority of patients.

To date, there are only a few other available pieces of data supporting the effectiveness of leflunomide as a steroid-sparing agent in GCA from a few other single-center studies, case series and case reports. A study carried out in Norway reported 11 retrospectively identified patients with difficult-to-treat GCA receiving leflunomide showing a significant reduction of CRP (*p* = 0.02) and a significantly smaller dose of prednisolone (*p* = 0.02) as early as after 3 months of treatment (17). Another retrospective Norwegian study, comparing leflunomide and methotrexate in the treatment of GCA, showed a significant difference in the time-to-remission rate in patients treated with leflunomide (56.4 vs. 86.4 weeks for leflunomide and methotrexate, respectively) (16). A case series from the UK demonstrated that 22 out of 23 patients (9 with difficult-to-treat GCA and 14 with difficult-to-treat polymyalgia rheumatica) had a complete or partial response to leflunomide, which was well-tolerated in all except in three patients, who experienced rashes, diarrhea and peritoneal abscesses (21).

Most of the up-to-date published studies are retrospective in nature and dealt with patients with difficult-to-treat diseases, some of whom had previously been unsuccessfully treated with another csDMARD (e.g., methotrexate), and who mostly required higher doses of glucocorticoids than the standard tapering regimen. It is therefore difficult to establish conclusions. The two prospective studies demonstrate additional evidence, but are limited by the relatively low number of patients included. Nevertheless, all the available data suggest the effectiveness of adjunctive treatment with leflunomide in GCA patients.

Our study was limited by its single-center, open-label design and the smaller size of the control group (glucocorticoid-only group); however, it was the result of a previously acquired positive experience with the use of leflunomide at our center. Due to its limitations, the results and conclusions of our study should be interpreted with caution. Nevertheless, we have presented the largest cohort of GCA patients treated with leflunomide to date. The main strength of our study is its prospective nature and external validity by means of the prospective inclusion of an unselected real-world GCA population, and the fact it followed a predefined systematic treatment regimen and follow-up strategy. Despite the limitations, this study significantly contributes to the growing knowledge of the effectiveness of leflunomide and its steroid-sparing effect in patients with GCA.

In conclusion, in this prospective single-center, open-label study, by adding leflunomide to the EULAR-recommended glucocorticoid regimen in GCA treatment, we demonstrated the encouraging potential of leflunomide to safely and effectively reduce the relapse rate at a lower cumulative glucocorticoid dose in over three quarters of GCA patients in our cohort.

Our experiences with leflunomide should be further verified in a randomized control trial.

## Data availability statement

The datasets analysed in the current study are available from the corresponding author upon reasonable request.

## Ethics statement

The studies involving human participants were reviewed and approved by Republic of Slovenia National Medical Ethics Committee, Ljubljana, Slovenia. The patients/participants provided their written informed consent to participate in this study.

## Author contributions

JK and AH contributed to the acquisition of data. AH analyzed and interpreted data. MT and ŽR helped revise the manuscript. All authors have read and approved the manuscript for submission.

## Funding

This study was funded by the Slovenian National Research grant number P3-0314.

## Conflict of interest

The authors declare that the research was conducted in the absence of any commercial or financial relationships that could be construed as a potential conflict of interest.

## Publisher's note

All claims expressed in this article are solely those of the authors and do not necessarily represent those of their affiliated organizations, or those of the publisher, the editors and the reviewers. Any product that may be evaluated in this article, or claim that may be made by its manufacturer, is not guaranteed or endorsed by the publisher.
